# Computer analysis of regulation of hepatocarcinoma marker genes hypermethylated by HCV proteins

**DOI:** 10.18699/VJGB-22-89

**Published:** 2022-12

**Authors:** E.A. Antropova, T.M. Khlebodarova, P.S. Demenkov, A.S. Venzel, N.V. Ivanisenko, A.D. Gavrilenko, T.V. Ivanisenko, A.V. Adamovskaya, P.M. Revva, I.N. Lavrik, V.A. Ivanisenko

**Affiliations:** Institute of Cytology and Genetics of the Siberian Branch of the Russian Academy of Scences, Novosibirsk, Russia; Institute of Cytology and Genetics of the Siberian Branch of the Russian Academy of Scences, Novosibirsk, Russia Kurchatov Genomic Center of ICG SB RAS, Novosibirsk, Russia; Institute of Cytology and Genetics of the Siberian Branch of the Russian Academy of Scences, Novosibirsk, Russia Kurchatov Genomic Center of ICG SB RAS, Novosibirsk, Russia; Institute of Cytology and Genetics of the Siberian Branch of the Russian Academy of Scences, Novosibirsk, Russia Kurchatov Genomic Center of ICG SB RAS, Novosibirsk, Russia; Institute of Cytology and Genetics of the Siberian Branch of the Russian Academy of Scences, Novosibirsk, Russia Kurchatov Genomic Center of ICG SB RAS, Novosibirsk, Russia; Institute of Cytology and Genetics of the Siberian Branch of the Russian Academy of Scences, Novosibirsk, Russia Novosibirsk State University, Novosibirsk, Russia; Institute of Cytology and Genetics of the Siberian Branch of the Russian Academy of Scences, Novosibirsk, Russia Kurchatov Genomic Center of ICG SB RAS, Novosibirsk, Russia; Kurchatov Genomic Center of ICG SB RAS, Novosibirsk, Russia Novosibirsk State University, Novosibirsk, Russia; Kurchatov Genomic Center of ICG SB RAS, Novosibirsk, Russia Kurchatov Genomic Center of ICG SB RAS, Novosibirsk, Russia; Translational Inflammation Research, Medical Faculty, Otto von Guericke University Magdeburg, Magdeburg, Germany; Institute of Cytology and Genetics of the Siberian Branch of the Russian Academy of Scences, Novosibirsk, Russia Kurchatov Genomic Center of ICG SB RAS, Novosibirsk, Russia Novosibirsk State University, Novosibirsk, Russia

**Keywords:** hepatocellular carcinoma, hepatitis C virus, expression regulation, methylation, regulatory pathways, gene networks, bioinformatics, гепатоцеллюлярная карцинома, вирус гепатита С, регуляция экспрессии, гиперметилирование, регуляторные пути, генные сети, биоинформатика

## Abstract

Hepatitis C virus (HCV) is a risk factor that leads to hepatocellular carcinoma (HCC) development. Epigenetic changes are known to play an important role in the molecular genetic mechanisms of virus-induced oncogenesis. Aberrant DNA methylation is a mediator of epigenetic changes that are closely associated with the HCC pathogenesis and considered a biomarker for its early diagnosis. The ANDSystem software package was used to reconstruct and evaluate the statistical significance of the pathways HCV could potentially use to regulate 32 hypermethylated genes in HCC, including both oncosuppressor and protumorigenic ones identified by genome-wide analysis of DNA methylation. The reconstructed pathways included those affecting protein-protein interactions (PPI), gene expression, protein activity, stability, and transport regulations, the expression regulation pathways being statistically significant. It has been shown that 8 out of 10 HCV proteins were involved in these pathways, the HCV NS3 protein being implicated in the largest number of regulatory pathways. NS3 was associated with the regulation of 5 tumor-suppressor genes, which may be the evidence of its central role in HCC pathogenesis. Analysis of the reconstructed pathways has demonstrated that following the transcription factor inhibition caused by binding to viral proteins, the expression of a number of oncosuppressors (WT1, MGMT, SOCS1, P53) was suppressed, while the expression of others (RASF1, RUNX3, WIF1, DAPK1) was activated. Thus, the performed gene-network reconstruction has shown that HCV proteins can influence not only the methylation status of oncosuppressor genes, but also their transcriptional regulation. The results obtained can be used in the search for pharmacological targets to develop new drugs against HCV-induced HCC.

## Abstract (ru)

Вирус гепатита С (ВГС) считается фактором риска для возникновения гепатоцеллюлярной карциномы (ГЦК). Известно, что большую роль в молекулярно-генетических механизмах вирус-индуцированного онкогенеза играют эпигенетические изменения. Аберрантное метилирование ДНК служит медиатором эпигенетических изменений, которые тесно связаны с патогенезом ГЦК, и признано биомаркером для его ранней диагностики. С помощью ANDSystem проведены реконструкция и оценка статистической значимости путей потенциальной регуляции вирусными белками ВГС 32 генов человека, гиперметилированных при ГЦК. Среди исследованных генов были как онкосупрессоры, так и проопухолевые гены, идентифицированных по данным полногеномного анализа метилирования ДНК. Реконструированы регуляторные пути, включающие белок-белковые взаимодействия, регуляцию экспрессии генов, регуляцию активности, стабильности и транспорта белков. Среди статистически значимых оказались пути регуляции экспрессии. Показано, что восемь из десяти белков ВГС являются участниками данных путей. Белок ВГС NS3 был вовлечен в наибольшее число регуляторных путей. NS3 связан с регуляцией пяти генов-онкосупрессоров, что может свидетельствовать о его центральной роли в патогенезе ГЦК. Анализ реконструированных путей показал, что при ингибировании транскрипционных факторов в результате связывания с вирусными белками, экспрессия ряда онкосупрессоров (WT1, MGMT, SOCS1, P53) подавлялась, тогда как экспрессия других (RASF1, RUNX3, WIF1, DAPK1) активировалась. Таким образом, с помощью реконструкции генных сетей показано, что вирусные белки гепатита С способны влиять не только на статус метилирования генов-онкосупрессоров, но и на их транскрипционную регуляцию. Полученные результаты могут быть использованы при поиске фармакологических мишеней для разработки новых средств против ГЦК, индуцированной ВГС.

## Introduction

Liver cancer is the third leading cause of cancer-related death in the world according to year 2020 statistics with over 900,000 new cases of this pathology registered the same year around the world (International Agency for Research on Cancer, https://gco.iarc.fr/today/home). Hepatocellular carcinoma (HCC) has been the dominant type of primary liver cancer, comprising about 90 % of all the cases (Llovet et al., 2016). It may be caused by several risk factors such as aflatoxin exposure, alcohol consumption; hepatitis B or C (HCV) virus infection, liver cirrhosis, non-alcoholic fatty liver disease, non-alcoholic steatohepatitis, metabolic syndrome, obesity, type II diabetes, and genetic predisposition (McGlynn et al., 2021).

Currently, a lot of data has been accumulated on HCV association with impaired liver function, cirrhosis and HCC development (Rabaan et al., 2020). Having gotten into a human body, HCV seeks to exercise control over the biological processes occurring in host cells in order to increase its survival and replication efficiency. In more than 70 % of those initially infected, the disease takes on a chronic course, so the patients experience progressive liver-tissue fibrosis and cirrhosis accompanied by long-term inflammation (Jaroszewicz et al., 2015). Using various mechanisms for infected cell cooptation, the virus can inadvertently lead to HCC development (D’souza et al., 2020). At the same time, the molecular and genetic mechanisms of virus-induced carcinogenesis remain understudied.

In addition, HCC pathogenesis is associated with epigenetic modifications and aberrant DNA methylation being a mediator of epigenetic changes (Fernández-Barrena et al., 2020) that can serve as a biomarker for early HCC diagnosis (Zhang C. et al., 2016; Xu et al., 2017).

To establish the functional links between genes and to elucidate the molecular mechanisms of biological processes, the methods for gene networks reconstruction have been widely employed. Previously, we developed the Associative Network Discovery System (ANDSystem) software package designed to reconstruct gene networks based on the knowledge extracted from factual databases and scientific publications using text-mining techniques (Ivanisenko V.A. et al., 2015, 2019; Ivanisenko T.V. et al., 2020). The package has enabled one to reconstruct the molecular mechanisms of a number of pathologies such as preeclampsia (Glotov et al., 2015), tuberculosis (Bragina et al., 2016), comorbid conditions of asthma and hypertension (Saik et al., 2018), COVID-19 (Ivanisenko N.V. et al., 2020), HCV life cycle (Saik et al., 2016), etc.

In the present study, ANDSystem was employed to reconstruct the regulatory pathways describing the potential regulation mechanisms of the genes hypermethylated in HCC by HCV proteins. The analysis looked at the 32 genes known to be hypermethylated HCC markers. Among the 7 types of reconstructed regulatory pathways including protein-protein interactions (PPI), gene expression, protein activity, stability and transport regulations, those responsible for gene expression regulation turned out to be statistically significant. Nine marker genes were identified that could potentially be subject to regulation by HCV proteins, including three HCC suppressor genes (MGMT, SOCS1 and TP53) that could be negatively regulated and one apoptosis suppressor gene (TERT ) that can be positively regulated.

## Materials and methods

**Fig. 1.**
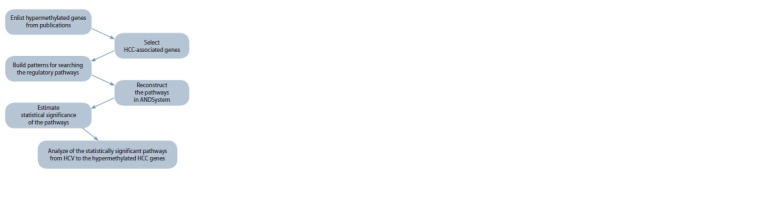
Schematic description of the data processing algorithm

Genes hypermethylated in HCC. Information about the hypermethylated genes was taken from publications (Table 1). Only those genes were considered whose hypermethylation was associated with HCC and confirmed through the analysis and meta-analysis given in the publications. The schematic of the data-processing algorithm can be seen in Figure 1.

**Table 1.**
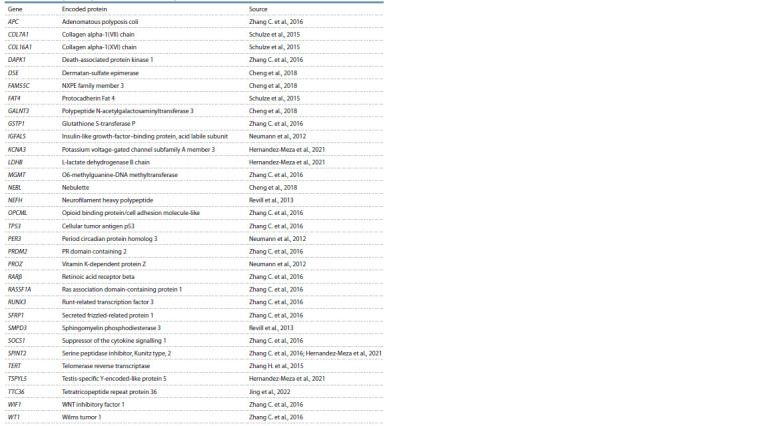
List of the hypermethylated genes used in the analysis

Regulatory pathways reconstruction in ANDSystem. The regulatory pathways were reconstructed using the ANDSystem software package (Ivanisenko V.A. et al., 2019) that had been designed to perform gene-networks reconstruction based on automated analysis of scientific texts and factual databases. ANDSystem includes a knowledge base with more than 40 million facts about molecular-genetic interactions, containing physical intermolecular interactions, gene expression, protein activity, stability and transport regulations. In the package, it is the the ANDVisio program that reconstructs and analyzes gene networks using the Pathway Wizard function performing search calls to the knowledge base according to a given pattern. A schematic description of the used patterns is given in Table 2

**Table 2.**
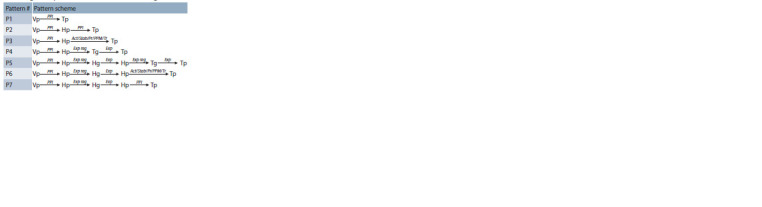
Patterns to search for the regulatory pathways describing viral – protein modulation of HCC marker genes Notе. Vp – HCV proteins; Hp – any human proteins involved in the interactions; Hg – any human genes involved in the interactions; Tg – target genes (HCC marker genes); Tp – Tg-encoded target genes; PPI – protein-protein interactions; Act/Stab/Pr/PPM/Tr – activity or stability regulation, or proteolysis, or post-translational modifications, or transport regulation function; Exp reg – gene-expression regulation; Exp – protein-producing gene expression.

For instance, P4 means searching for all possible molecular genetic pathways in the ANDSystem knowledge base that satisfy the following requirement: the first participant in the pathway is the viral protein (Vp); the second is human protein (Hp); the third is a human gene from a list of target genes (Tg); the last member of the pathway is a Tg-encoded protein (Tp). Further in the text, HCC marker genes will be regarded as target ones. Interactions between pathway participants are represented by the following types: Vp and Hp are linked by PPIs; Hp and Tg are “expression regulation” interaction type (Exp reg), where Hp regulates Tg gene expression; Tg and Tp are interaction of the “expression” interaction type (Exp), i. e., the Tp protein is the expression product of the Tg gene. Examples of regulatory pathway reconstruction in ANDSystem using the patterns presented in the previous work (Ivanisenko V.A. et al., 2022).

Estimating the statistical significance of pathways. The pattens from Table 2 were used to calculate the number of marker genes K participating in the regulatory pathways, as well as the number of such participants in the sample of control gene. The likelihood of observing the number K for random reasons was estimated using the standard hypergeometric distribution and the hypergeom function from the SciPy 1.8.0 package (https://scipy.org). For the purposes of statistical processing, a group of genes proposed by Hoshida et al. (2008) as a control to predict HCC outcomes based on the expression level of genes was taken

## Results

Reconstruction of the potential regulatory pathways HCV proteins use to affect HCC marker genes

A set of hypermethylated HCC marker genes was used to reconstruct the potential regulatory pathways through which viral proteins could modulate the genes playing an important role in HCC pathogenesis (see Table 1). The set had been based on the published results of a genome-wide analysis of DNA methylation and included 30 genes, the expression of which, according to the studies, was reduced in hepatocellular carcinoma, and two genes (WT1 and TERT ) with increased expression

To reconstruct the regulatory pathways, the ANDSystem software package was used. The search queries to the knowledge base were formed using the pathway patterns presented in Table 2. The patterns described different types of regulatory pathways determined by different combinations of moleculargenetic interactions, including PPIs, gene expression, protein activity, stability, and transport regulations

Analysis of the statistical significance of the pathways automatically reconstructed by ANDSystem according to the given patterns showed that among the seven types of regulatory pathways analyzed, expression regulation ones turned out to be statistically significant (P4 in Table 3). This pattern describes the pathways including four participants: (1) viral proteins; (2) human transcription factors (TF) involved in PPIs with viral proteins; (3) marker genes presented in Table 1, whose expression is regulated by (2); (4) protein products of marker genes.

**Table 3.**
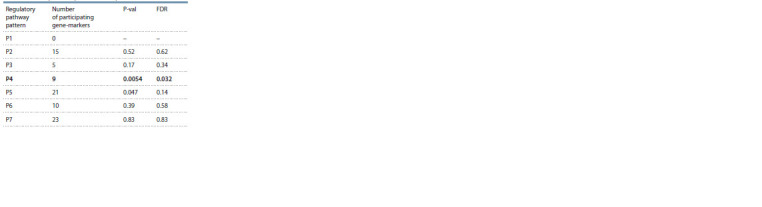
Results of assessing the significance of the regulatory pathways described by different patterns Notе. P-val is the level of statistical significance; FDR is the level of statistical significance accounting for multiple comparisons as per the false discovery rate (expected proportion of false rejections).

**Fig. 2.**
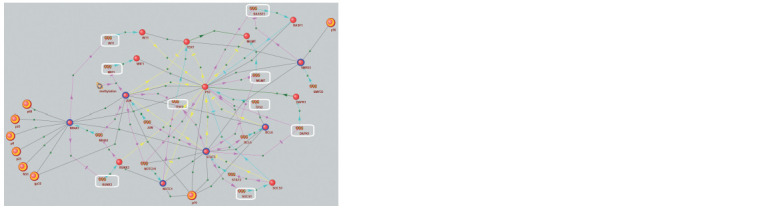
Gene network including statistically significant regulatory pathways for the viral proteins to influence HCC marker genes expression that was reconstructed in ANDSystem using the P4 pattern. Legend: HCV proteins (yellow-red large balls) – p8 (Non-structural protein 4A, NS4A), p21 (Core, Capsid protein C), p23 (Protease NS2-3), gp32 (Envelope glycoprotein E1), NS1 (Envelope glycoprotein E2), p56 (NS5A), p68 (NS5B), p70 (Hepacivirin, NS3); intermediate proteins (blue-red balls) – BCL6 (B-cell lymphoma 6 protein), NOTC1 (Neurogenic locus notch homolog protein 1), NR4A1 (Nuclear receptor subfamily 4 group A member 1), JUN (c-Jun/activator protein 1), SMYD3 (lysine methyltransferase SET and MYND domain containing protein 3), STAT3 (Signal transducer and activator of transcription 3); hypermethylated genes (highlighted in white frames) and their protein products – DAPK1 (Death associated protein kinase 1), MGMT (Methylated-DNA-protein-cysteine methyltransferase), RASSF1 (Ras association domain family member 1), RUNX3 (Runt-related transcription factor 3), SOCS1 (Suppressor of cytokine signaling 1), TERT (Telomerase reverse transcriptase), TP53 (Tumor protein p53), WIF1 (Wnt inhibitory factor 1), WT1 (Wilms tumor protein).

The gene network describing the regulation pathways of HCC marker genes included 8 HCV proteins, 7 intermediate host proteins involved in PPIs with HCV proteins, and 9 genes (DAPK1, SOCS1, MGMT, RASSF1, RUNX3, TP53, WIF1, WT1, and TERT) whose aberrant expression correlated with HCC progression (Fig. 2).

Analysis of the regulatory pathways

The regulatory pathways involved 8 out of 10 HCV proteins and 6 human genes, which protein products acting as the intermediate participants the viral proteins could form protein heterocomplexes with. The latter included such genes of transcription factors as STAT3 (Signal transducer and activator of transcription 3), NR4A1 (Nuclear receptor subfamily 4 group A member 1), JUN (c-Jun/activator protein 1), BCL6 (B-cell lymphoma 6 protein), transmembrane receptor NOTC1 (Neurogenic locus notch homolog protein 1) and histone methyltransferase SMYD3 (Lysine methyltransferase SET and MYND domain containing protein 3).

Most of the viral proteins were associated with RUNX3 and WT1 regulation. Six of them (NS4A, Core, p23, gp32, NS1, and NS5B) interacted with NR4A1 being a general expression regulator of these two HCC marker genes.

HCV protein NS3 (p70) interacted with the largest number of expression regulators, and through these interactions it could potentially regulate the expression of five tumor suppressor genes and that of TERT.

Now, let us consider the possibilities of implementing of these regulatory pathways in more detail.

p8, p21, p68, gp32, p23, NS1/NR4A1/RUNX3, WT1. This regulatory pathway suggests six HCV proteins (p8, p21, p68, gp32, p23, NS1) can possibly affect HCC development by controlling the activity of the RUNX3 and WT1 genes through the NR4A1 transcription factor. Indeed, NR4A1 directly interacts with the RUNX3 and WT1 promoters, suppressing RUNX3 activity and activating that of WT1 (Nowyhed et al., 2015; Zong et al., 2017). Both factors are involved in apoptosis regulation, hence, RUNX3 promotes activation of the extrinsic, TRAIL-induced apoptosis pathway (Kim et al., 2019), while WT1 controls the mitochondrial (internal) apoptosis pathway through the regulation of the Bcl-2 anti-apoptotic protein gene, and, depending on a cell type, affects the expression of the Bcl-2 gene in both positive and negative ways (Mayo et al., 1999; Loeb, 2006). It has been shown that in HCC, an increased expression of the WT1 gene is observed, which is due to hypermethylation of its promoter and correlates with a poor prognosis (Sera et al., 2008; Mžik et al., 2016). These data suggest that the role WT1 plays in HCC progression is associated with blocked apoptosis.

Experiments have demonstrated that HCV core protein inhibits at least the NR4A1 and RUNX3 genes expression in infected cells (Tan, Li, 2015), contributing to suppressing an external apoptosis pathway. The Y2H test (Two Hybrid Test) has shown NR4A1 can interact with such viral proteins as CORE, E1, E2, NS2, NS4A, and NS5B (de Chassey et al., 2008), but except for CORE, the effects of the other HCV proteins on TF activity have not been investigated yet.

E1, NS3, Core, p23, NS1, p68/JUN, NOTC1, STAT3/ TERT. Aberrant expression of the TERT gene, associated, among other things, with hypermethylation of its promoter, is a prognostic marker of HCC (Zhang H. et al., 2015; Zucman- Rossi et al., 2015; Oversoe et al., 2020). TERT affects disease progression through stimulation of cell proliferation due to reactivation of its gene expression in carcinoma cells (Nault et al., 2019; In der Stroth et al., 2020). TERT activity has been shown to also increase in HCV-infected cells, partly through direct interaction of the core protein with the enzyme (Zhu et al., 2010, 2017), however, in general, the mechanisms HCV proteins affect TERT activity are not clear. This regulatory pathway suggests the involvement of the virus NS3, Core, E1, p23, NS1, and NS5B proteins in TERT gene expression through interaction with the JUN (AP-1), STAT3, and NOTC1 proteins

Indeed, experiments have shown that there is a possibility to affect TERT expression through the AP-1 and STAT3 TFs being its direct regulators (Konnikova et al., 2005; Takakura et al., 2005), as well as through the NOTC1 signaling pathway (Sawangarun et al., 2018). Moreover, it has been demonstrated that the HCV NS3 protein affects NOTC1 activity through the SRCAP transcription factor (Iwai et al., 2011) and the expression of AP1- and STAT3-regulated genes (Hassan et al., 2005, 2007; Machida et al., 2006; Li et al., 2010), however, the specific mechanisms of realization of these influences in infected hepatocyte cells have not been practically examined.

gp32, p70/JUN/WIF1. This regulatory pathway describes the effect the NS3 and E1 HCV proteins have on WIF1 (Wnt inhibitory factor 1) gene expression through interaction with the c-Jun/AP-1 TF. WIF1 is a tumor suppressor that reduces cell growth in HCC (Deng et al., 2010), and its expression level is a prognostic indicator of the course of the disease (Huang et al., 2011).

Experiments have demonstrated that there is both the possibility of a direct effect of the NS3 and E1 proteins on the activity of c-Jun/AP-1 (de Chassey et al., 2008), and the latter can affect the expression of the WIF1 gene through interaction with the DNMT1 methyltransferase (DNA methyltransferase 1), suppressing WIF1 in gallbladder cancer cells (Lin et al., 2018). But what mechanisms of WIF1 gene suppression are initiated in HCV-infected hepatocarcinoma cells remains unknown.

p70/STAT3/MGMT, DAPK1, SOCS1. This regulatory pathway is initiated by NS3 (p70) affecting the activity of the MGMT, SOCS, and DAPK1 genes through interaction with the STAT3 TF. Proteins DAPK1 (Death-associated protein kinase 1), MGMT (Methylated-DNA-protein-cysteine methyltransferase), and SOCS1 (Suppressor of cytokine signaling 1) are considered tumor suppressors, and their low expression in carcinomas correlates with disease progression (Gui et al., 2011; Jiang et al., 2019; Chen J. et al., 2020; Chen P. et al., 2020; Song et al., 2020).

Experiments have demonstrated that NS3 can directly interact with the STAT3 TF (de Chassey et al., 2008) involved in the regulation of the expression of the abovementioned genes (Kohsaka et al., 2012; Benderska, Schneider-Stock, 2014; Yang C. et al., 2015), however, the effect of STAT3 on MGMT, SOCS, and DAPK1 expression is not unambiguous and may be associated with cell specialization. As for the mechanisms regulating the expression of these genes in HCV-infected hepatocarcinoma cells, they have not been studied yet.

p70/BCL6/TP53. TP53 is a key activator of intrinsic apoptosis pathway. The NS3 (p70) protein affects TP53 through interaction with the BCL6 (B-cell lymphoma 6 protein) TF. TP53 is a HCC marker gene of and its low expression correlates with poor disease prognosis (Liu et al., 2012; Ye et al., 2017). BCL6 represses the TP53 gene in lymphoid cells, and its constitutive expression protects B lymphocytes from DNA damage-induced apoptosis (Phan, Dalla-Favera, 2004; Jardin et al., 2007). However, the data describing the effect of HCV has on TP53 and BCL6 expression in these cells are associated with a possible mutation induction and are mutually exclusive (Machida et al., 2004; Tucci et al., 2013). The interaction of NS3 and BCL6 is discussed in (Han et al., 2016), but the specific mechanisms NS3 affects the TF activity have not been studied yet.

## Discussion

**Fig. 3.**
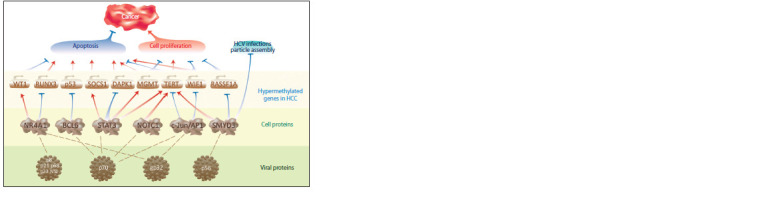
Interrelation of the reconstructed regulatory pathways and the key biological processes associated with HCC.

The studied set of hypermethylated HCC marker genes (see Table 1) included 30 downexpressed and two over-expressed genes. Using ANDSystem, the regulatory pathways by which HCV proteins are able to influence the expression of these marker genes were reconstructed. The relationship between the pathways and HCC-associated key biological processes is shown in Figure 3. According to the published data, the WT1, RUNX3, TP53, and SOCS1 genes are closely associated with apoptosis (Mayo et al., 1999; Loeb, 2006; Kim et al., 2019), while the MGMT, TERT, RASSF1A, and WIF1 genes – with apoptosis and cell proliferation (He et al., 2005; Sarin et al., 2005; Choi et al., 2008; Feng et al., 2014; Chen J. et al., 2020; Ni et al., 2020).

The analysis showed the identified pathways could potentially be a part of the mechanism HCV proteins affect the activity of HCC marker genes. However, the effects the proteins have on the function of human regulatory proteins during PPI formation are currently poorly understood. This fact prevents us from unambiguous interpretation of the reconstructed pathways, because what determines if a regulatory pathway functions as an activator or suppressor of target gene expression is whether or not the ability to regulate gene expression remains in the host regulatory protein after its interaction with the viral protein. Investigation of these effects requires further experimental studies and computer molecular modeling.

The literature describes the effects viral proteins can have on the function of host proteins, e. g., the NS5A protein is known to bind to SMYD3 in the cytoplasm and inhibit SMYD3 translocation to the nucleus (Chen M. et al., 2016). If the regulatory proteins of a host organism are assumed to lose their ability to regulate gene expression due to the complexes formed with viral proteins, then the following effects can be expected: when considering the pathways regulating onco-suppressor expression, four of the seven pathways inhibiting RASF1, RUNX3, WIF1, and DAPK1 will be suppressed, which can lead to their activation by HCV proteins, and that, in turn, will prevent carcinogenesis. In the remaining three pathways, the activation of MGMT, SOCS1 and TP53 will be suppressed, which may possibly have a protumor effect. In the presented pathways (see Fig. 2), WT1, MGMT, SOCS1 and TP53 are activated by the corresponding factors (Phan et al., 2004; Kohsaka et al., 2012; Yang C. et al., 2015; Zong et al., 2017), while the expression of RASF1, RUNX3, WIF1, and DAPK1 genes is negatively controlled (Guo et al., 2011; Benderska, Schneider-Stock, 2014; Nowyhed et al., 2015; Lin et al., 2018).

In the case of the TERT and WT1 genes that can be attributed to those with protumor activity, suppression of the WT1 gene can be expected and will lead to a negative effect on carcinogenesis. As for the TERT gene involved in apoptosis suppression and stimulating cell proliferation (Nault et al., 2019; In der Stroth et al., 2020), according to our results (see Fig. 2), this gene was controlled through three different regulatory pathways. Its expression was activated by two pathways involving the STAT3 and NOTC1 host genes (Konnikova et al., 2005; Sawangarun et al., 2018), and one of the pathways suppressed the expression involving c-JUN/AP-1 (Takakura et al., 2005). The interaction with these host proteins could lead to a blockage of these regulatory pathways. The pathway involving c-JUN/AP-1 may be of particular interest in this respect, since inhibition of this TF by viral proteins (gp32 and NS3) could promote TERT activation. These assumptions are in good agreement with the data on differential gene expression in acute HCV infection when the infected cells showed an increased TERT expression (Papic et al., 2012), so this pathway can be a promising pharmacological target.

Thus, the considered assumptions lead one to conclude the presence of multidirectional regulation of the observed expression of HCC marker genes. This may indicate that not all regulatory pathways controlled by viral proteins can be attributed to HCC risk factors. However, the regulatory pathways that ensure the protumor activity of virus proteins undoubtedly deserve additional study to understand the mechanisms of virus-induced HCC carcinogenesis. In particular, the suppression of tumor suppressor gene expression by viral proteins can enhance the effect of their methylation in HCC or mimic this effect when these genes are not methylated, which can either provoke HCC onset or complicate its course.

## Conclusion

Using the computer methods for gene network reconstruction available in the ANDSystem package, the statistically significant pathways for HCV proteins to regulate HCC gene markers have been established. The obtained results describe the potential mechanisms of the proteins involvement in HCC pathogenesis and may be useful for planning experimental studies to search for new targets for the development of drugs and prophylactic agents to reduce the risk of HCC developing in presence of HCV infection

## Conflict of interest

The authors declare no conflict of interest.
